# Gene delivery ability of polyethylenimine and polyethylene glycol dual-functionalized nanographene oxide in 11 different cell lines

**DOI:** 10.1098/rsos.170822

**Published:** 2017-10-25

**Authors:** Liping Wu, Jinshan Xie, Tan Li, Zihao Mai, Lu Wang, Xiaoping Wang, Tongsheng Chen

**Affiliations:** 1 MOE Key Laboratory of Laser Life Science, College of Biophotonics, South China Normal University, Guangzhou, Guangdong, People's Republic of China; 2 Department of Pain Management, The First Affiliated Hospital of Jinan University, Guangzhou, Guangdong, People's Republic of China

**Keywords:** polyethylenimine, polyethylene glycol, graphene oxide, gene delivery, transfection efficiency

## Abstract

We recently developed a polyethylenimine (PEI) and polyethylene glycol (PEG) dual-functionalized reduced graphene oxide (GO) (PEG−nrGO−PEI, RGPP) for high-efficient gene delivery in HepG2 and Hela cell lines. To evaluate the feasibility and applicability of RGPP as a gene delivery carrier, we here assessed the transfection efficiency of RGPP on gene plasmids and siRNA in 11 different cell lines. Commercial polyalkyleneimine cation transfection reagent (TR) was used as comparison. In HepG2 cells, RGPP exhibited much stronger delivery ability for siRNA and large size plasmids than TR. For green fluorescent protein (GFP) plasmid, RGPP showed about 47.1% of transfection efficiency in primary rabbit articular chondrocytes, and about 27% of transfection efficiency in both SH-SY5Y and A549 cell lines. RGPP exhibited about 37.2% of GFP plasmid transfection efficiency in EMT6 cells and about 26.0% of GFP plasmid transfection efficiency in LO2 cells, but induced about 33% of cytotoxicity in both cell lines. In 4T1 and H9C2 cell lines, RGPP had less than 10% of GFP plasmid transfection efficiency. Collectively, RGPP is a potential nano-carrier for high-efficiency gene delivery, and needs to be further optimized for different cell lines.

## 1. Introduction

Gene delivery provides a powerful tool for exploring gene function, generating transgenic organisms and treating gene-related diseases [1–3]. A variety of gene functional sensors have been designed to monitor intracellular dynamic processes such as activation of protein kinases, oligomerization of proteins and protein–protein interactions in single living cells [4–10]. Although RNA interference can only partially silence a target gene and has extensive off-target effects, it has been widely used for genome-wide loss-of-function screens and evaluating the function of genes and proteins [11–15]. CRISPR/Cas9, a versatile genome editing technology, can target nearly any DNA sequence, and the high efficiency of genome editing with Cas9 makes it possible to alter many targets in parallel, thereby enabling unbiased genome-wide functional screens to identify genes that play an important role in a phenotype of interest [15–17]. Moreover, CRISPR/Cas9 can generate cellular transgenic models for studying human polygenic diseases, generating transgenic animal models and repressing or activating gene transcription [15,18–23]. With high potential to treat diseases at genetic roots, gene therapy has long fascinated scientists and clinicians. In the past two decades, a series of phase I/II gene-therapy clinical trials have been reported to have significant efficacy and safety for the treatment of various severe inherited diseases of the immune, blood and nervous systems [24–28].

Safe and efficient carrier is critical for gene delivery [29]. Polyethylenimine (PEI), a cationic polymer with the highest cationic-charge-density potential, is a potential excellent carrier of gene delivery [30,31]. PEI can efficiently bind anionic DNA/RNA within the physiological pH range, protect DNA/RNA from degradation and trigger DNA/RNA release from endosome [32–37]. In contrast to the high cytotoxicity and poor biocompatibility of PEI, PEI-functionalized nanographene oxide (nGO−PEI) composites have an improved transfection efficiency with lower cytotoxicity [30,38–40]. Polyethylene glycol (PEG) can further improve the biocompatibility of nGO−PEI in the presence of saline or serum [41–45]. Moreover, PEG and PEI dual-functionalized GO (GO-PEI-PEG) also exhibited excellent transfection capacity and low cytotoxicity [42–48]. Recently, we developed a PEI and PEG dual-functionalized reduced GO (PEG−nrGO−PEI, RGPP) composite for high-efficiency gene delivery, exhibiting about 83.9% of transfection efficiency for green fluorescent protein (GFP) plasmid in Hela cells [45].

In this report, we evaluate the delivery ability of RGPP for siRNA and plasmids in 11 kinds of cell lines. Commercial polyalkyleneimine cation transfection reagent (TR) was used as comparison. In HepG2 cells, RGPP exhibited better gene delivery ability than TR for siRNA, large size for plasmids such as GFP-Bcl-xL (9.8 kb) and other functional gene plasmids including GFP-Bak, GFP-Puma, GFP-Bax 1–2/L-6 and GFP-NPM NLS1/2D. RGPP could efficiently deliver GFP plasmid into primary rabbit articular chondrocyte, SH-SY5Y, A549, EMT6 and LO2 cell lines. However, RGPP inefficiently delivered GFP plasmid into H9C2 and 4T1 cell lines.

## 2. Material and methods

### 2.1. Materials

GO was purchased from Nanjing XFNano Materials Tech Co. Ltd (Nanjing, China). Branched PEI (25 kDa) and *N*-(3-dimethylaminopropyl-*N*′-ethylcarbodiimide) hydrochloride (EDC) were purchased from Sigma-Aldrich (St Louis, USA). 8-arm-polyethylene glycol amine (10 kDa, PEG-NH_2_) was purchased from Shanghai Seebio Biotech, Inc. (Shanghai, China). Turbofect™ transfection regent (commercial polyalkyleneimine cation TR) was purchased from Thermo Fisher Scientific (Massachusetts, USA). Cell Counting Kit-8 (CCK-8) was purchased from Dojindo Laboratories (Kumamoto, Japan). Dulbecco's modified Eagle's medium (DMEM) and RPMI 1640 medium were purchased from Gibco (Grand Island, USA). Fetal bovine serum (FBS) was purchased from Hangzhou Sijiqing Biological Engineering Materials Co. Ltd (Hangzhou, China).

Human hepatoma cell line HepG2 was purchased from the Experimental Animal Center of Sun Yat-Sen University (Guangzhou, China). Human neuroblastoma cell line SH-SY5Y, human breast cancer cell line MCF-7, mouse melanoma cell line B16 and high metastatic mouse melanoma cell line B16F10 were obtained from American Type Culture Collection. Human normal hepatocyte cell line LO2, human lung cancer cell line A549, mouse breast cancer cell line EMT6 and high metastatic mouse breast cancer cell line 4T1 were obtained from Jinan University (Guangzhou, China). Rat myocardial cell line H9C2 was obtained from Guangdong Provincial People's Hospital (Guangzhou, China). Primary rabbit articular chondrocytes were prepared according to our previously reported method and identified by using toluidine blue staining [49,50].

Venus (V, #27794), C32 V (Cerulean-32-Venus, #26396), VCV (Venus-Cerulean-Venus, #27788), VCVV (Venus-Cerulean-Venus-Venus, #27789), EGFP-Bak (#32564), EGFP-Puma (#16590), EGFP-Bax 1-2/L-6 (#30533), EGFP-Bcl-xL (GBX, #64123), GFP-Bcl2 (#17999), GFP-Bcl2-Cb5 (#18000), GFP-Bcl2-Maob (#18001), GFP-NPM WT (#17578), GFP-NPM NESD (#13283), GFP-NPM NLS1/2D (#13287) and EGFP (#74165) plasmids were obtained from Addgene (Cambridge, MA, USA). FAM-labelled siRNA was purchased from GenePharma (Shanghai, China).

### 2.2. Synthesis of PEG−nGO

GO was prepared by a modified Hummers method using expandable graphite flake [51,52]. GO (10 mg) was sonicated with Ultrasonic Cell Crusher (JY92-2D; Xinzhi Ultrasonic Equipment Co, Ningbo, China) for 1 h in ice bath to obtain nano-GO (nGO) suspension. NaOH (1.2 g) and ClCH_2_COOH (1.0 g) were added to the nGO suspension and sonicated for 30 min in ice bath to obtain carboxylation of nGO (nGO−COOH). The resulting nGO−COOH suspension was washed three to five times with deionized water by using 30 kDa molecular weight cut off (MWCO) ultrafiltration device (Millipore, Bedford, MA, USA) to remove ions.

PEG−nGO was obtained just as described previously [53]. Briefly, 2 ml of EDC aqueous solution (2.0 mg ml^−1^) was added to 10 ml of nGO suspension (1.0 mg ml^−1^), and pH of the mixture was adjusted to 8.0 by 5 mM NaOH and then sonicated for another 10 min. Next, 30 mg of 8-arm PEG-NH_2_ was added to the above suspension, and the mixture was sonicated for 10 min and then stirred for 12 h at room temperature to obtain nGO–PEG. The resulting nGO–PEG suspension was washed three to five times with deionized water by using ultrafiltration device (30 kDa MWCO) to remove the unreacted PEG and ions.

### 2.3. Synthesis of PEG−nrGO−PEI

RGPP was prepared as described previously [45]. Briefly, 2 ml PEG–nGO solution (1.0 mg ml^−1^) was mixed with 8 ml PEI (25 kDa) solution (0.75 mg ml^−1^) and then bathed at 80°C for 2 h to obtain RGPP solution. The resulting RGPP solution was dialysed against deionized water in a 30 kDa MWCO dialysis membrane for 2 days. The 630 nm ultraviolet–visible (UV–vis) absorbance of cuprammonium complex formed between PEI and copper(II) ion was measured by UV–vis spectrometer (Lambda 35; PerkinElmer, MA, USA) for determining the amount of modified PEI in RGPP [54].

### 2.4. Cell culture and cytotoxicity assay

HepG2, SH-SY5Y, MCF-7, B16, B16F10, A549 cells and primary rabbit articular chondrocytes were cultured in high-glucose DMEM supplemented with 10% FBS. H9C2 cells were cultured in low-glucose DMEM containing 10% FBS. LO2, EMT6 and 4T1 cells were cultured in RPMI 1640 containing 10% FBS. All cells were maintained in a humidified incubator with 5% CO_2_ at 37°C.

Cytotoxicity of RGPP and TR as well as plasmid was assessed by CCK-8 assay according to the manufacturer's protocol just as described previously [55]. Cells were collected and seeded in 96-well plates with a density of 1 × 10^4^ cells well^−1^ and incubated for 24 h. Plasmid (0.2 µg) and different amount of RGPP or TR were mixed in 20 µl serum-free media and incubated at room temperature for 20 min. Plasmid/RGPP or plasmid/TR complexes were incubated with cells for 4 h in 100 µl serum-free media. Then, serum-free media were replaced by fresh media containing 10% FBS and the cells were incubated for another 44 h before CCK-8 assay.

### 2.5. Transfection assay of plasmids or siRNA

Cells were seeded in 24-well plates with a density of 1 × 10^5^ cells/well and incubated for 24 h before transfection. For siRNA transfection, 0.369 µg of FAM-labelled siRNA and different amount of RGPP or TR were mixed in 100 µl serum-free media and incubated at room temperature for 20 min. Complexes of RGPP/siRNA or TR/siRNA were incubated with cells for 4 h in 500 µl serum-free media, and then siRNA transfection efficiency was determined by fluorescence microscope imaging (Olympus IX73 equipped with a CCD camera, Japan) and flow cytometry analysis (FCM, FACSCCanto II, BD Biosciences, NJ, USA). For plasmid transfection, 1 µg of plasmid and different amount of RGPP or TR were mixed in 100 µl serum-free media and incubated at room temperature for 20 min. After the complexes of RGPP/plasmid or TR/plasmid were incubated with cells for 4 h in 500 µl serum-free media, the serum-free media were replaced by fresh media containing 10% FBS and the cells were cultured for another 44 h before fluorescence microscope imaging or FCM analysis for transfection efficiency.

### 2.6. Statistics

Data were presented as mean ± s.d. from at least three independent experiments. The Student's *t*-test was used to evaluate the significance of difference between two groups. Statistical and graphic analyses were done using the software SPSS 19.0 (SPSS, Chicago) and Origin 8.5 (OriginLab Corporation). *p* < 0.05 was defined as statistically significant difference.

## 3. Results and discussion

### 3.1. Transfection efficiency of RGPP on plasmids and siRNA in HepG2 cells

**Figure 1.**
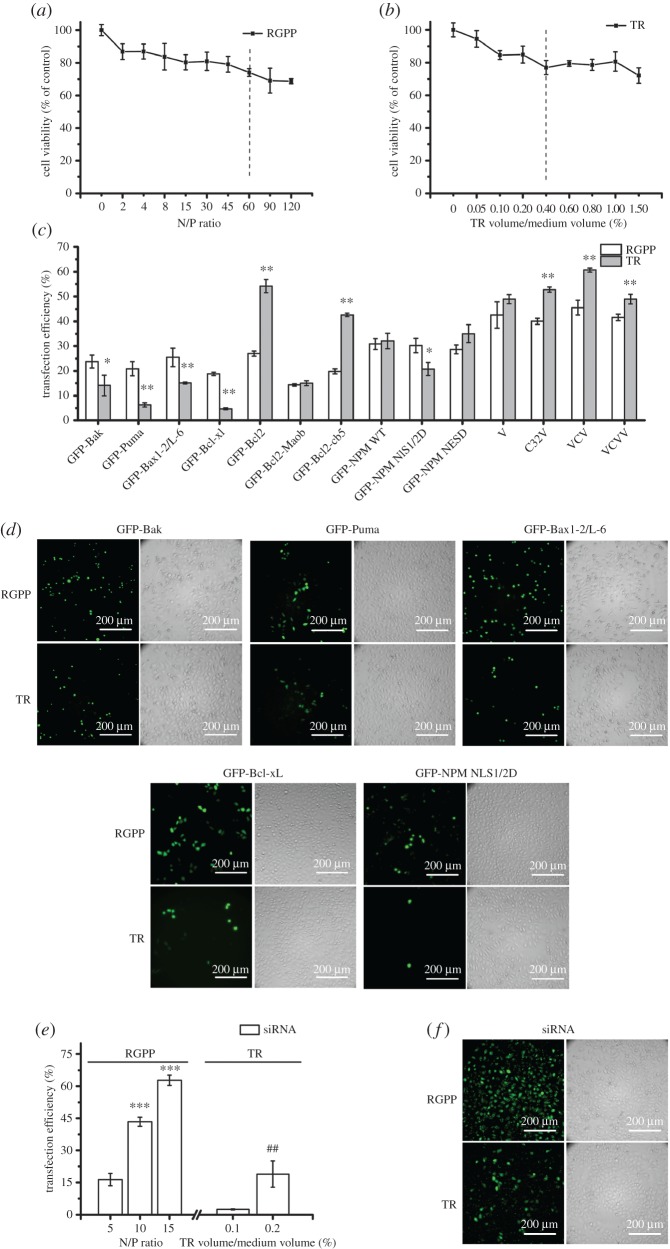
Transfection efficiency of RGPP on plasmids and siRNA in HepG2 cells. (*a,b*) Relative cell viability of cells treated with different concentrations of RGPP or TR for 48 h determined by CCK-8 assay. (*c*) Transfection efficiency of RGPP or TR for various plasmids after transfection for 48 h determined by FCM analysis. *p < 0.05 and **p < 0.01, compared with RGPP group. (*d*) Fluorescence microscopic images of cells exclusively transfected with GFP-Bak, GFP-Puma, GFP-Bax 1-2/L-6, GFP-Bcl-xL and GFP-NPM NLS1/2D plasmids by using RGPP at N/P ratio of 60 or 0.4% of TR for 48 h. Scale bar, 200 µm. (*e*) Transfection efficiency of RGPP or TR on siRNA after transfection for 4 h determined by FCM analysis. ***p < 0.001, compared with RGPP at N/P ratio of 5; ^##^*p* < 0.01, compared with 0.1% of TR. (*f*) Fluorescence microscopic images of cells transfected with siRNA by using RGPP at N/P ratio of 15 or 0.2% of TR for 4 h. Scale bar, 200 µm.

After exposure of cells to RGPP or TR for 48 h, CCK-8 assay showed that both RGPP and TR exhibited dose-dependent cytotoxicity, and RGPP at N/P ratio of 60 and 0.4% (TR volume/medium volume) of TR had similar cytotoxicity (figure 1*a,b*). RGPP at N/P ratio of 60 and 0.4% of TR were used for plasmid transfection without indication.

We evaluated the transfection ability of RGPP/TR for 10 functional gene plasmids by using FCM analysis. FCM analysis showed that RGPP had higher transfection efficiency than TR for GFP-Bak (23.8 ± 2.6% for RGPP, 14.2 ± 4.2% for TR), GFP-Puma (20.9 ± 2.9% for RGPP, 6.3 ± 0.9% for TR), GFP-Bax 1-2/L-6 (25.5 ± 3.7% for RGPP, 15.1 ± 0.3% for TR), GFP-Bcl-xL (18.9 ± 0.6% for RGPP, 4.7 ± 0.4% for TR) and GFP-NPM NLS1/2D (30.3 ± 2.9% for RGPP, 20.8 ± 2.6% for TR) plasmids (figure 1*c*). Fluorescence microscopic images of cells exclusively transfected GFP-Bak, GFP-Puma, GFP-Bax 1-2/L-6, GFP-Bcl-xL and GFP-NPM NLS1/2D plasmids by using RGPP and TR for 48 h also demonstrated that RGPP showed higher delivery ability for these five plasmids than TR (figure 1*d*). We also determined the transfection ability of both RGPP and TR for V, C32 V, VCV and VCVV plasmids, and found that both RGPP and TR efficiently delivered these plasmids into cells, and the transfection ability of TR was slightly better than that of RGPP (figure 1*c*). After the complexes of RGPP/siRNA or TR/siRNA were incubated with cells for 4 h, FCM analysis showed that RGPP at N/P ratios of 10 and 15 exhibited 43.4 ± 2.1% and 62.8 ± 2.4% of siRNA transfection efficiency, much higher than that of 0.1% and 0.2% of TR (figure 1*e*). Fluorescence microscopic images also showed that RGPP at N/P ratio of 15 delivered more siRNA into cells than 0.2% of TR (figure 1*f*).

Size of plasmid is one of the elements influencing transfection efficiency during transient transfection assay [56,57]. Transfection efficiency of RGPP for GFP-Bcl-xL plasmid (9.8 kb) was about 18.9%, about threefold higher than TR (about 4.7%; figure 1*c*), suggesting that RGPP exhibited better delivery ability for large-size plasmid than TR. It was reported that huge aggregates (greater than 1000 nm) of DNA/transferrin-PEI exhibited much higher transfection efficiency than small aggregates in *in vitro* transfection assay [58]. Diameter of the GFP-Bcl-xL plasmid/RGPP complexes was about 987 nm, and the particle size of DNA/TR was 50–150 nm [59], which might be the reason why RGPP more efficiently delivered large-size plasmid into cells than TR. In addition, RGPP also exhibited better delivery ability for siRNA than TR (figure 1*e,f*), which might benefit from the inhibitory effect of RGPP on gene degradation [30].

### 3.2. Transfection efficiency of RGPP on GFP plasmid in human cancer cell lines

**Figure 2.**
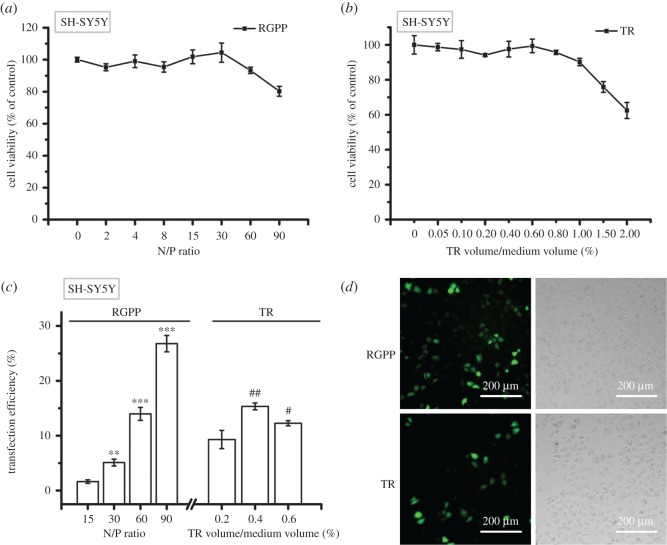
Transfection efficiency of RGPP on GFP plasmid in SH-SY5Y cells. (*a,b*) Relative cell viability of cells treated with different concentrations of RGPP or TR for 48 h determined by CCK-8 assay. (*c*) Transfection efficiency of RGPP or TR on GFP plasmid after transfection for 48 h determined by FCM analysis. **p < 0.01 and ***p < 0.001, compared with RGPP at N/P ratio of 15; ^#^*p* < 0.05 and ^##^*p* < 0.01, compared with 0.2% of TR. (*d*) Fluorescence microscopic images of cells transfected with GFP plasmid by using RGPP at N/P ratio of 90 or 0.4% of TR for 48 h. Scale bar, 200 µm.

We evaluated the delivery ability of RGPP on GFP plasmid by using FCM analysis in three human cancer cell lines: human neuroblastoma cell line (SH-SY5Y), human lung cancer cell line (A549) and human breast cancer cell line (MCF-7). Figure 2*a,b* shows the cytotoxicity of different doses of RGPP or TR in SH-SY5Y cells, and RGPP at N/P ratios of 15, 30, 60 and 90, respectively, exhibited 2.1 ± 0.3%, 5.1 ± 0.6%, 14.0 ± 1.2% and 27.2 ± 1.5% of transfection efficiency, while 0.2%, 0.4% and 0.6% of TR, respectively, showed 9.3 ± 1.7%, 15.0 ± 0.6% and 12.3 ± 0.5% of transfection efficiency (figure 2*c*). RGPP at N/P ratio of 60 and 0.4% of TR exhibited similar cytotoxicity (about 4.5%) and transfection efficiency (about 14.0%) in SH-SY5Y cells (figure 2*a–c*). RGPP at N/P ratio of 90 showed the highest transfection efficiency (about 27.2%), about 1.8-fold that of 0.4% of TR (about 15.0%; figure 2*c*), suggesting that RGPP at N/P ratio of 90 was superior to 0.4% of TR as a gene delivery carrier for SH-SY5Y cells. Fluorescence microscopic images also showed that RGPP at N/P ratio of 90 delivered more GFP plasmid into SH-SY5Y cells than 0.4% of TR (figure 2*d*).

The cytotoxicity and transfection efficiency of RGPP and TR in A549 and MCF-7 cells are listed in table 1. In A549 cells, RGPP at N/P ratios of 15, 30, 60 and 90, respectively, exhibited 9.7 ± 1.2%, 15.0 ± 1.0%, 27.3 ± 1.0% and 45.4 ± 3.6% of transfection efficiency with 13.2 ± 0.1%, 13.1 ± 2.4%, 17.8 ± 4.5% and 33.3 ± 8.5% of cytotoxicity, and 0.2% and 0.4% of TR showed 5.2 ± 0.4% and 30.1 ± 2.0% of transfection efficiency without cytotoxicity, and 0.6% of TR exhibited 18.0 ± 2.1% of transfection efficiency with 10.0 ± 2.0% of cytotoxicity (table 1). In MCF-7 cells, RGPP at N/P ratios of 15, 30 and 60, respectively, exhibited 5.5 ± 0.2%, 7.0 ± 1.0% and 11.4 ± 1.8% of transfection efficiency with 5.2 ± 4.6%, 7.7 ± 1.0% and 10.0 ± 5.2% of cytotoxicity, while 0.2%, 0.4% and 0.6% of TR, respectively, had 21.4 ± 0.3%, 18.9 ± 0.6% and 16.0 ± 1.0% of transfection efficiency with 14.1 ± 8.4%, 17.4 ± 6.6% and 14.3 ± 5.2% of cytotoxicity (table 1).

**Table 1.** Transfection efficiency (TE) of RGPP on GFP plasmid in A549 and MCF-7 cell lines (CV, cell viability).

		RGPP (N/P ratio)				TR (TR volume/medium volume) (%)		
		15	30	60	90	0.2	0.4	0.6
A549	CV (%)	86.8 ± 0.1	86.9 ± 2.4	82.2 ± 4.5	66.7 ± 8.5	99.9 ± 3.7	95.4 ± 4.3	90.0 ± 2.0
	TE (%)	9.7 ± 1.2	15.0 ± 1.0	27.3 ± 1.0	45.4 ± 3.6	5.2 ± 0.4	30.1 ± 2.0	18.0 ± 2.1
MCF-7	CV (%)	94.8 ± 4.6	92.3 ± 1.0	90.0 ± 5.2	79.1 ± 4.0	85.9 ± 8.4	82.6 ± 6.6	85.7 ± 5.2
	TE (%)	5.5 ± 0.2	7.0 ± 1.0	11.4 ± 1.8	16.6 ± 0.1	21.4 ± 0.3	18.9 ± 0.6	16.0 ± 1.0

### 3.3. Transfection efficiency of RGPP on GFP plasmid in mouse cancer cell lines

**Figure 3.**
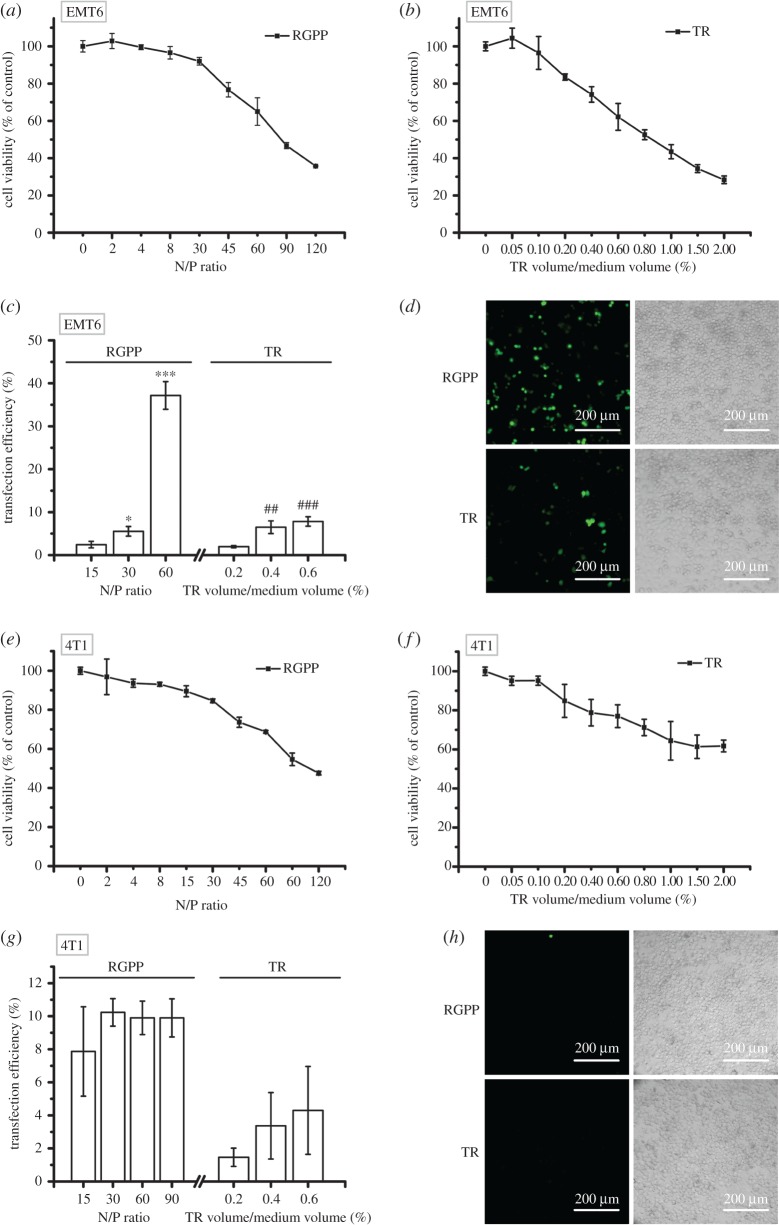
Transfection efficiency of RGPP on GFP plasmid in mouse cancer cell lines. (*a,b*) Relative cell viability of EMT6 cells treated with different concentrations of RGPP or TR for 48 h determined by CCK-8 assay. (*c*) Transfection efficiency of RGPP or TR on GFP plasmid after transfection for 48 h in EMT6 cells determined by FCM analysis. *p < 0.05 and ***p < 0.001, compared with RGPP at N/P ratio of 15; ^##^*p* < 0.01 and ^###^*p* < 0.001, compared with 0.2% of TR. (*d*) Fluorescence microscopic images of EMT6 cells transfected with GFP plasmid by using RGPP at N/P ratio of 60 or 0.6% of TR for 48 h. Scale bar, 200 µm. (*e,f*) Relative cell viability of 4T1 cells treated with different concentrations of RGPP or TR for 48 h determined by CCK-8 assay. (*g*) Transfection efficiency of RGPP or TR on GFP plasmid after transfection for 48 h in 4T1 cells determined by FCM analysis. (*h*) Fluorescence microscopic images of 4T1 cells transfected with GFP plasmid by using RGPP at N/P ratio of 30 or 0.6% of TR for 48 h. Scale bar, 200 µm.

We next evaluated the delivery ability of RGPP on GFP plasmid in four mouse cancer cell lines: mouse breast cancer cell line (EMT6), high metastatic mouse breast cancer cell line (4T1), mouse melanoma cell line (B16) and high metastatic mouse melanoma cell line (B16F10). As shown in figure 3*a,b*, both RGPP and TR exhibited dose-dependent cytotoxicity in EMT6 cells, and RGPP at N/P ratios of 15, 30 and 60, respectively, had 2.4 ± 0.8%, 5.5 ± 1.1% and 37.2 ± 3.2% of transfection efficiency, and 0.2%, 0.4% and 0.6% of TR, respectively, had 2.0 ± 0.3%, 6.5 ± 1.5% and 7.8 ± 1.1% of transfection efficiency (figure 3*c*). In EMT6 cells, RGPP at N/P ratio of 60 and 0.6% of TR exhibited about 35% of cytotoxicity, but transfection efficiency of RGPP at N/P ratio of 60 (about 37.2%) was about fourfold higher than that of 0.6% of TR (about 7.8%; figure 3*a–d*). Similarly in 4T1 cells, both RGPP and TR also exhibited dose-dependent cytotoxicity (figure 3*e,f*), and RGPP at N/P ratios of 15, 30, 60 and 90, respectively, exhibited 7.9 ± 2.7%, 10.2 ± 0.8%, 9.9 ± 1.0% and 9.9 ± 1.2% of transfection efficiency, and 0.2%, 0.4% and 0.6% of TR, respectively, had 1.5 ± 0.6%, 3.4 ± 2.0% and 4.3 ± 2.7% of transfection efficiency (figure 3*g*). Both RGPP and TR showed less than 10% of transfection efficiency indicating that both RGPP and TR were inefficient in delivering plasmid into 4T1 cells. Fluorescence microscopic images also showed that both RGPP and TR were inefficient in delivering plasmid into 4T1 cells (figure 3*h*).

The cytotoxicity and transfection efficiency of RGPP and TR in B16F10 and B16 cells are listed in table 2. In B16F10 cells, RGPP at N/P ratios of 15, 30, 60 and 90, respectively, exhibited 8.7 ± 1.4%, 9.3 ± 0.7%, 18.1 ± 1.8% and 32.4 ± 1.3% of transfection efficiency with 17.3 ± 6.0%, 29.2 ± 4.6%, 36.9 ± 1.9% and 44.1 ± 1.2% of cytotoxicity, and 0.2%, 0.4% and 0.6% of TR, respectively, had 31.8 ± 1.9%, 33.7 ± 0.9% and 25.9 ± 0.1% of transfection efficiency with 20.3 ± 1.9%, 40.4 ± 3.2% and 40.9 ± 2.8% of cytotoxicity (table 2). In B16 cells, RGPP at N/P ratios of 30, 60 and 90, respectively, exhibited 3.4 ± 0.2%, 17.0 ± 0.1% and 32.8 ± 4.1% of transfection efficiency with 3.3 ± 9.1%, 9.5 ± 3.9% and 39.9 ± 5.4% of cytotoxicity, while 0.2%, 0.4% and 0.6% of TR, respectively, exhibited 4.3 ± 3.2%, 19.7 ± 0.4% and 16.2 ± 0.9% of transfection efficiency without cytotoxicity (table 2). Therefore, compared with RGPP, TR was more suitable as a gene delivery carrier in B16F10 (0.2% of TR) and B16 (0.4% of TR) cell lines.

**Table 2.** Transfection efficiency (TE) of RGPP on GFP plasmid in B16F10 and B16 cell lines (CV, cell viability).

		RGPP (N/P ratio)				TR (TR volume/medium volume) (%)		
		15	30	60	90	0.2	0.4	0.6
B16F10	CV (%)	82.7 ± 6.0	70.8 ± 4.6	63.1 ± 1.9	55.9 ± 1.2	79.7 ± 1.9	59.6 ± 3.2	59.1 ± 2.8
	TE (%)	8.7 ± 1.4	9.3 ± 0.7	18.1 ± 1.8	32.4 ± 1.3	31.8 ± 1.9	33.7 ± 0.9	25.9 ± 0.1
B16	CV (%)	108.0 ± 13.8	96.7 ± 9.1	90.5 ± 3.9	60.1 ± 5.4	106.5 ± 21.4	104.2 ± 12.6	116.5 ± 33.6
	TE (%)	2.1 ± 0.4	3.4 ± 0.2	17.0 ± 0.1	32.8 ± 4.1	4.3 ± 3.2	19.7 ± 0.4	16.2 ± 0.9

### 3.4. Transfection efficiency of RGPP on GFP plasmid in normal cells

**Figure 4.**
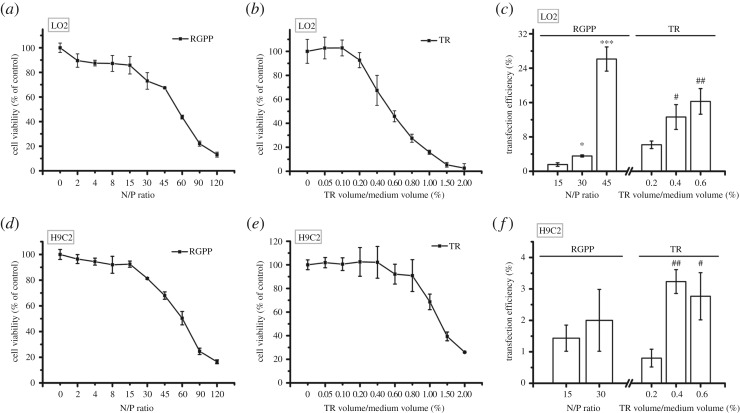
Transfection efficiency of RGPP on GFP plasmid in LO2 cells and H9C2 cells. (*a,b*) Relative cell viability of LO2 cells treated with different concentrations of RGPP or TR for 48 h determined by CCK-8 assay. (*c*) Transfection efficiency of RGPP or TR on GFP plasmid after transfection for 48 h in LO2 cells determined by FCM analysis. *p < 0.05 and ***p < 0.001, compared with RGPP at N/P ratio of 15; ^#^*p* < 0.05 and ^##^*p* < 0.01, compared with 0.2% of TR. (*d,e*) Relative cell viability of H9C2 cells treated with different concentrations of RGPP or TR for 48 h determined by CCK-8 assay. (*f*) Transfection efficiency of RGPP or TR on GFP plasmid after transfection for 48 h in H9C2 cells determined by FCM analysis. ^#^*p* < 0.05 and ^##^*p* < 0.01, compared with 0.2% of TR.

**Figure 5.**
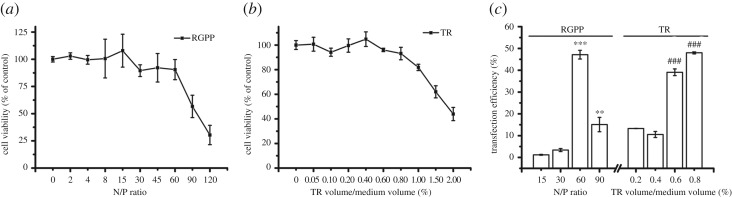
Transfection efficiency of RGPP on GFP plasmid in primary rabbit articular chondrocyte. (*a,b*) Relative cell viability of rabbit articular chondrocyte treated with different concentrations of RGPP or TR for 48 h determined by CCK-8 assay. (*c*) Transfection efficiency of RGPP or TR on GFP plasmid after transfection for 48 h in rabbit articular chondrocyte determined by FCM analysis. **p < 0.01 and ***p < 0.001, compared with RGPP at N/P ratio of 15; ^###^*p* < 0.001, compared with 0.2% of TR.

Finally, we evaluated the delivery ability of RGPP on GFP plasmid in normal cells including human normal hepatocyte cell line (LO2), rat myocardial cell line (H9C2) and primary rabbit articular chondrocytes. In LO2 cells, RGPP at N/P ratios of 15, 30 and 45, respectively, exhibited 1.6 ± 0.4%, 3.6 ± 0.3% and 26.1 ± 2.8% of transfection efficiency with 14.2 ± 7.1%, 27.0 ± 6.7% and 32.6 ± 0.3% of cytotoxicity, and 0.2%, 0.4% and 0.6% of TR, respectively, showed 6.2 ± 0.9%, 12.6 ± 2.9% and 16.3 ± 3.0% of transfection efficiency with 7.3 ± 6.3%, 32.6 ± 12.5% and 54.2 ± 4.6% of cytotoxicity (figure 4*a–c*). Both RGPP at N/P ratio of 45 and 0.4% of TR had about 32.6% of cytotoxicity, but the transfection efficiency of RGPP at N/P ratio of 45 was about 26.1%, about twofold that of 0.4% of TR (about 12.6%) in LO2 cells (figure 4*a–c*). In H9C2 cells, RGPP at N/P ratios of 15 and 30, respectively, exhibited only 1.4 ± 0.4% and 2.0 ± 1.0% of transfection efficiency with 7.5 ± 2.4% and 18.7 ± 0.6% of cytotoxicity, and 0.2% and 0.4% of TR exhibited 0.8 ± 0.3% and 3.2 ± 0.4% of transfection efficiency without cytotoxicity, and 0.6% of TR had 2.8 ± 0.8% of transfection efficiency with 7.9 ± 8.4% of cytotoxicity (figure 4*d–f*). Transfection efficiencies of RGPP and TR were less than 4% (figure 4*f*), indicating that both RGPP and TR were inefficient in delivering plasmid into H9C2 cells. In primary rabbit articular chondrocytes, RGPP at N/P ratios of 15, 30, 60 and 90, respectively, had 1.2 ± 0.2%, 3.4 ± 0.7%, 47.1 ± 2.0% and 15.1 ± 3.3% of transfection efficiency, and 0.2%, 0.4%, 0.6% and 0.8% of TR, respectively, exhibited 13.3 ± 0.1%, 10.5 ± 1.4%, 39.0 ± 1.5% and 48.0 ± 0.5% of transfection efficiency (figure 5*c*). RGPP at N/P ratio of 60 and 0.8% of TR exhibited similar cytotoxicity (about 8.5%) and transfection efficiency (47.1 ± 2.0% for RGPP, 48.0 ± 0.5% for TR) in primary rabbit articular chondrocytes (figure 5*a–c*). Therefore, both RGPP at N/P ratio of 60 and 0.8% of TR were excellent gene delivery carriers for primary rabbit articular chondrocytes.

## 4. Conclusion

The transfection efficiency of RGPP for GFP plasmid in 10 different cell lines summarized in table 3 demonstrates that RGPP is a potential gene delivery carrier. Especially, RGPP can efficiently deliver siRNA and large-size plasmids. RGPP exhibits efficient plasmid delivery ability in primary rabbit articular chondrocyte, SH-SY5Y, A549, EMT6 and LO2 cell lines. Moreover, based on the excellent photothermal efficiency of reduced GO (rGO), NIR enhances gene delivery ability of RGPP. Collectively, RGPP is a potential nano-carrier for high-efficiency gene delivery and further studies are needed to optimize its gene delivery ability for different cell lines.

**Table 3.** Cytotoxicity and transfection efficiency (TE) of RGPP and TR at optimum dose in ten different cell lines. Cytotoxicity = 1 − (cell viability); RAC, rabbit articular chondrocytes.

	RGPP			TR		
cell line	N/P ratio	cytotoxicity (%)	TE (%)	TR volume/medium volume (%)	cytotoxicity (%)	TE (%)
SH-SY5Y	90	20.0 ± 3.1	27.2 ± 1.5	0.4	3.0 ± 4.5	15.0 ± 0.6
A549	60	18.0 ± 4.5	27.3 ± 1.0	0.4	4.6 ± 4.3	30.1 ± 2.0
MCF-7	90	20.0 ± 4.0	16.6 ± 0.1	0.2	14.1 ± 8.4	21.4 ± 0.3
EMT6	60	34.0 ± 7.4	37.2 ± 3.2	0.6	38.0 ± 7.2	7.8 ± 1.1
4T1	30	19.0 ± 1.0	10.2 ± 0.8	0.6	25.0 ± 5.8	4.3 ± 2.7
B16F10	60	37.0 ± 1.9	18.1 ± 1.8	0.2	20.3 ± 1.9	31.8 ± 1.9
B16	60	9.5 ± 3.9	17.0 ± 0.1	0.4	−4.2 ± 12.6	19.7 ± 0.4
LO2	45	32.6 ± 0.3	26.0 ± 2.8	0.6	54.2 ± 4.6	16.3 ± 3.0
H9C2	30	20.0 ± 0.6	2.0 ± 1.0	0.4	−2.0 ± 13.4	3.2 ± 0.4
RAC	60	9.5 ± 9.3	47.1 ± 2.0	0.8	8.0 ± 5.1	48.0 ± 0.5

## Supplementary Material

Raw data of cell viability in response to cytotoxicity of RGPP or TR.

## Supplementary Material

Raw data of transfection efficiency by transfection reagent RGPP or TR.

## Data accessibility

Raw data for experiments are found in the electronic supplementary material.
